# Entropy in Cell Biology: Information Thermodynamics of a Binary Code and Szilard Engine Chain Model of Signal Transduction

**DOI:** 10.3390/e20080617

**Published:** 2018-08-19

**Authors:** Tatsuaki Tsuruyama

**Affiliations:** Department of Discovery Medicine, Pathology Division, Graduate School of Medicine, Kyoto University, Yoshida-Konoe-cho, Sakyo-ku, Kyoto 606-8315, Japan; tsuruyam@kuhp.kyoto-u.ac.jp; Tel.: +81-75-366-4694; Fax: +81-75-334-6252

**Keywords:** biological reaction cascade, binary code system, average entropy production rate, mutual entropy, Szilard engine chain, fluctuation theorem

## Abstract

A model of signal transduction from the perspective of informational thermodynamics has been reported in recent studies, and several important achievements have been obtained. The first achievement is that signal transduction can be modelled as a binary code system, in which two forms of signalling molecules are utilised in individual steps. The second is that the average entropy production rate is consistent during the signal transduction cascade when the signal event number is maximised in the model. The third is that a Szilard engine can be a single-step model in the signal transduction. This article reviews these achievements and further introduces a new chain of Szilard engines as a biological reaction cascade (BRC) model. In conclusion, the presented model provides a way of computing the channel capacity of a BRC.

## 1. Introduction

Information science provides a theoretical framework for understanding cell biology. Variable types of information entropy have been defined and applied for biological research. “Single cell entropy” was introduced for the estimation of the specified gene and kinase protein expression network [1,2]. Further, multicellular behaviour was analysed by a mathematical model in which individual cells interact with each other by secretion and sensing [3,4]. Immunological responses against variable antigens were quantified using entropy defined by the selective probability of amino acid residues [5]. The genetic entropy defined by DNA mutation rate is computable and useful in analysing molecular evolution [6], and the correlation analysis of the mutated gene frequency responsible for the cancer pattern development provides a useful predictive data for clinical prognosis. Further, the transfer entropy is generalised by the Kullback–Leibler divergence between two probabilistic transition statuses along a time course and a measurement of the transfer entropy enables the quantification of the information flow between stationary systems evolving in time [7,8]. Mutual entropy was defined on the basis of the correlation analysis between enzymes and metabolites such as ATP [9].

In addition to these recent develpments, significant achievements have been reported by the application of information thermodynamics to cell system that involves a feedback controller; hence, it can be an integrative system, in which information and thermodynamic entropy intersect [10,11,12,13,14,15]. Many reports on the study of information-driven works have recently been presented. For example, an information-driven artificial molecular motor device consisting of an enzyme has been reported [11].

The upper limit of the average work <*w*> that can be extracted from thermodynamic engine depends on the system temperature *T*, Boltzmann constant *k_B_*, free energy change Δ*F* and mutual entropy *H* informed by the feedback controller [12,13,14,15]:

(1) $$\langle w\rangle \leq -\Delta F+k_{B}TH.$$

Inequality (1) implies that free energy and mutual entropy are exchangeable parameters. For the isovolumic and isothermal biological system, Inequality (1) can be simplified to

(2) $$\langle w\rangle \leq k_{B}TH.$$

As shown in below, the work extracted from an ideal *Szilard* engine is,

(3) $$\langle w\rangle =k_{B}TH$$

From the viewpoint of information thermodynamics, the signal transduction in *Escherichia coli* was reported [16]. Our earlier works considered a probability that the mutual entropy may be utilised for exchanging signalling molecules along the biological reaction cascade (BRC) [17,18,19,20]. This review later particularly introduces an ideal chain of Szilard engine constituting the BRC model [17].

## 2. A Common BRC Model

Let us consider modelling signal transduction by focusing on aspects that are common to several signal transductions. In BRC, the substrate protein in the reaction may become an enzyme or modulator in the next reaction. The most well-known example of a chain reaction is the chain of phosphorylation of proteins in the mitogen-activated protein kinase (MAPK) cascade [21,22,23,24,25,26] that is shown in (4):

(4) $$\begin{array}{l} \begin{array}{l} EGF+EGFR(X_{1})↔EGF+EGFR*(X_{1}*),\\ EGFR*+Ras(X_{2})\to EGFR*+Ras*(X_{2}*),\\ Ras*+c-Raf(X_{3})\to Ras*+c-Raf*(X_{3}*), \end{array}\\ \begin{array}{l} c-Raf*+MEK(X_{4})\to c-Raf*+MEK*(X_{4}*),\\ MEK*+ERK(X_{5})\to MEK*+ERK*(X_{5}*). \end{array} \end{array}$$

In this BRC, the epidermal growth factor receptor (EGFR), Ras (a type of GTPase), a proto-oncogene c-Raf, MAP kinase-extracellular signal-regulated kinase (MEK) and kinase-extracellular signal-regulated kinase (ERK) follow the stimulation with the epidermal growth factor (EGF). Phosphatases were omitted in the above equation. This MAPK cascade is a ubiquitous signalling pathway in variable cell types, which allows growth and proliferation. The EGFR mutation promotes the enhancement of this cascade, which contributes to the tumourogenesis of the lung and other cancers [27].

To understand the essence of complicated cell signaling, the BRC model consisting of *j^th^* and reverse −*j^th^* steps can be constructed (1 ≤ *j* ≤ *n*):

(5) $$\begin{array}{l} X_{1}(R)+L\to X_{1}-L*:1^{st}\\ X_{1}-L*\to X_{1}+L:-1^{st}\\ X_{1}-L*+X_{2}+A\to X_{1}-L*+X_{2}*+D:2^{nd}\\ X_{2}*+Ph_{2}\to X_{2}+Ph_{2}+Pi:-2^{nd}\\ \cdots \\ X_{j}*+X_{j+1}+A\to X_{j}*+X_{j+1}*+D:j^{th}\\ X_{j+1}*+Ph_{j+1}\to X_{j+1}+Ph_{j+1}+Pi:-j^{th}\\ \cdots \\ X_{n-1}*+X_{n}+A\to X_{n-1}*+X_{n}*+D:{\left(n-1\right)}^{th}\\ X_{n}*+Ph_{n}\to X_{n}+Ph_{n}+Pi:-{\left(n-1\right)}^{th}\\ X_{n}*+DNA+RNApol+N\;ribonucleotide\to \\ X_{n}+DNA*+RNApol+{\left(ribonucleotide\right)}_{N}:n^{th} \end{array}$$

Each step represents an activation of the signalling molecules *X_j_* in the cytoplasm maintained by a chemical reservoir of mediator *A*, such as adenosine triphosphate (ATP), and an inactivation of the signalling molecules X_j_* by enzymes *Ph_j_*. ATP is hydrolysed into adenosine diphosphate (ADP; *D* in (5)) and inorganic phosphate (*Pi*), which modifies the amino acid residue of *X_j_*. *X_j_* and X_j_* denote unmodified (inactive) and modified (active) signalling molecules, respectively. The first reaction represents the ligand (*L*), EGF in the MAPK cascade, an extracellular molecule and stimulates *X*_1_, which represents a receptor (*R*), EGFR in the MAPK cascade, on the cellular membrane. Afterward, *X*_1_ − L* complex promotes the modification of *X*_2_ in the cytoplasm into *X*_2_* activated by *Pi* that originated from *A*, and *D* is produced. Further, *X*_2_* promotes the modification of *X*_3_ into *X*_3_*. In this manner, the *j^th^* signalling molecule, X_j_*, activates *X_j_*_+1_ in the cytoplasm into *X_j_*_+1_*. Following the (*n* − 1)*^th^* step, the signalling molecule X_n_* binds to the promoter region of the DNA and induces the *m*RNA transcription in the *n^th^* step. In addition, during the reverse BRC steps, the inactivation of X_j_* into *X_j_* occurs through enzymes that catalyse inactivation or through self-inactivation by X_j_*, in which *Pi* is released. Thus, *j^th^* and −*j^th^* step forms a cycle reaction consisting of activation and inactivation. Finally, a pre-stimulation steady state of the individual step is recovered. Such reaction chain schemes were previously described by Gaspard et al. and Tsuruyama [17,18,28,29,30].

## 3. Binary Code Model of BRCs

Recent studies showed that the BRC can be interpreted as a binary code system with two forms of signalling molecules, namely an active form (X_j_*) and an inactive form (*X_j_*) in individual step [18,19,20]. The total signal event number *Ψ* in a given BRC event, can be described using the concentration of inactive *X_j_* and active molecules X_j_* as follows:

(6) $$\Psi =\frac{X!}{\overset{n}{\underset{j=1}{\prod }}X_{j}!\overset{n}{\underset{j=1}{\prod }}X_{j}*!}$$

*X* represents the total concentration of signalling molecules. The logarithm of *Ψ* is approximated according to Starling’s equation and gives Shannon’s entropy *S* using the selection probability of *X_j_* or X_j_*, *p_j_* = *X_j_*/*X* and p_j_* = X_j_*/*X* [19]:

(7) $$S=\log\Psi =-X\left(\overset{n}{\underset{j=1}{\sum }}p_{j}\log p_{j}+\overset{n}{\underset{j=1}{\sum }}p_{j}*\log p_{j}*\right)$$

Selecting the *j^th^* step component of *S* in (7) gives:

(8) $$s_{j}≜-X\left[p_{j}\log p_{j}+p_{j}*\log p_{j}*\right]$$

When the signal is transmitted to the *j^th^* step, concentrations of *X_j_* or X_j_* fluctuate, and *s_j_* is given using the probability fluctuation *dp_j_* and dp_j_*,

(9) $$s_{j}≜-X\left[\left(p_{j}+dp_{j}\right)\log\left(p_{j}+dp_{j}\right)+\left(p_{j}*+dp_{j}*\right)\log\left(p_{j}*+dp_{j}*\right)\right]$$

Because the signal has not yet reached the (*j* + 1)*^th^* step; hence, the entropy of the (*j* + 1)*^th^* step remains:

(10) $$s_{j+1}≜-X\left[p_{j}\log p_{j}+p_{j}*\log p_{j}*\right]$$

Therefore, the entropy difference *H_j_* is generated between the *j^th^* and (*j* + 1)*^th^* step is presented as follows [17,18,19]:

(11) $$H_{j}≜s_{j}-s_{j+1}=X\Delta p_{j}*\log\frac{p_{j}}{p_{j}*}=\Delta X_{j}*\log\frac{p_{j}}{p_{j}*}$$

with

(12) $$X_{j}+X_{j}*=const.$$

(13) $$p_{j}+p_{j}*=const.$$

and

(14) $$\begin{array}{l} \Delta X_{j}+\Delta X_{j}*=0\\ \Delta p_{j}+\Delta p_{j}*=0 \end{array}$$

In addition, the entropy difference per single active molecule, *h_j_*, is given by Equation (11):

(15) $$h_{j}≜H_{j}/\Delta X_{j}*=\log\frac{p_{j}}{p_{j}*}$$

In previous report [19], entropy current *C_j_* was introduced as follows:

(16) $$C_{j}=k_{B}T\frac{\partial s_{j}}{\partial p_{j}*}\Delta p_{j}*\approx k_{B}T\log\frac{p_{j}}{p_{j}*}\Delta X_{j+1}*$$

Accordingly, the entropy current density *c_j_* per single active molecule is given as:

(17) $$c_{j}=\frac{C_{j}}{\Delta X_{j}*}=k_{B}T\log\frac{p_{j}}{p_{j}*}$$

## 4. Mutual Entropy in BRCs

**Figure 1:**
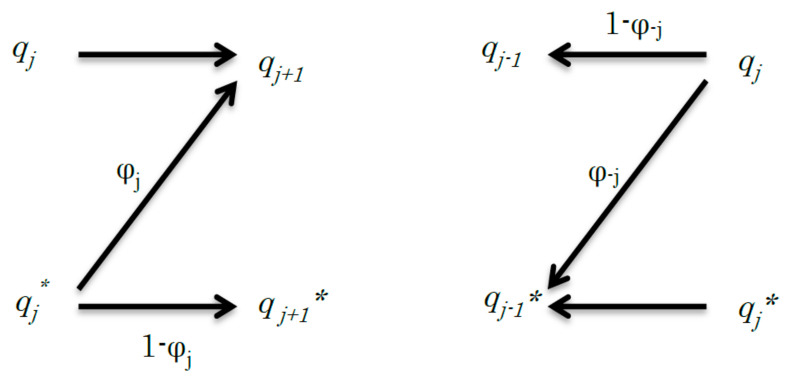
Schematic of the relationship between the *j^th^* step to (*j* + 1)*^th^* step (left) and the −*j^th^* step to (−*j* − 1)*^th^* step (right) of a simple discrete channel. The left graph shows a signal transduction and its channel capacity is expressed by *C_j_*. The right graph shows the reverse signal transduction and its channel capacity is expressed by *C*_−*j*_. In the reverse signal transduction, from the −*j^th^* step to (−*j* − 1)*^th^* step, *q_j_* transmits the signal to *q_j−_*_1_, but it may transmit the signal to *q_j−_*_1_*** in error.

For the evaluation of mutual entropy in Equation (15) according to information theory, let us consider the channel capacity of the *j^th^* cycle (1 ≤ *j* ≤ *n*) (Figure 1). The natural logarithm was applied in place of the base-2 logarithm to simplify the description. The entropy *h_j_*^0^ is given using *q_j_* = *p_j_*/(*p_j_* + p_j_*) and q_j_* = p_j_*/(*p_j_* + p_j_*), as follows:

(18) $$h_{j}{}^{0}=-q_{j}\log p_{j}-q_{j}*\log q_{j}*$$

The conditional entropies *h_j_* (*j* + 1|*j*) from the (*j* + 1)*^th^* step for the given *j^th^* step can be described as a linear function of q_j_* using the probability of the noise occurrence probability *ϕ_j_* and *ξ_j_* = −*ϕ_j_*log*ϕ_j_* − (1 − *ϕ_j_*)log (1 − *ϕ_j_*) [13]:

(19) $$h(j+1|j)=-\xi_{j}q_{j}*\equiv -\left(\varphi_{j}\log\varphi_{j}+(1-\varphi_{j})\log\varphi_{j}\right)q_{j}*$$

*h_j_*^0^ and *h*(*j* + 1|*j*) are chosen in such a manner as to maximize mutual entropy, which is defined by *h_j_*^0^ − *h*(*j* + 1|*j*), subject to the constraint q_j_* + *q_j_* = 1. The channel capacity is defined as the maximum value of mutual entropy:

(20) $$c_{j}≜{\left[h_{j}{}^{0}-h\left(j+1|j\right)\right]}^{\max}$$

with

(21) $$h_{j}≜h_{j}{}^{0}-h\left(j+1|j\right)$$

To obtain the maximized mutual entropy, the following function *U_j_* using the undetermined parameter *λ* is maximized using Lagrange’s method for undetermined multipliers as follows [13]:

(22) $$U_{j}=-q_{j}\log q_{j}-q_{j}*\log q_{j}*-\xi_{j}q_{j}*+\lambda \left[q_{j}+q_{j}*\right]$$

Then

(23) $$\frac{\partial }{\partial q_{j}}U_{j}=-\log q_{j}-1+\lambda$$

(24) $$\frac{\partial }{\partial q_{j}*}U_{j}=-\log q_{j}*-1+\lambda -\xi_{j}$$

Setting the right-hand side of Equations (23) and (24) to zero, and eliminating *λ*, we have:

(25) $$\log\frac{q_{j}}{q_{j}*}=\log\frac{p_{j}}{p_{j}*}=\xi_{j}$$

From *q_j_* + q_j_* = 1 and (25), the following can be derived:

(26) $$q_{j}=\frac{\phi_{j}}{\phi_{j}+1}$$

(27) $$q_{j}*=\frac{1}{\phi_{j}+1}$$

with

(28) $$\phi_{j}=\exp\left(\xi_{j}\right)$$

As a result, the channel capacity of the *j^th^* step, *C_j_*, is given using (21), (26)–(28) as a maximum value of mutua entropy:

(29) $$C_{j}≜h_{j}{}^{\max}=\left(-\frac{\phi_{j}}{\phi_{j}+1}\log\frac{\phi_{j}}{\phi_{j}+1}-\frac{1}{\phi_{j}+1}\log\frac{1}{\phi_{j}+1}-\xi_{j}\frac{\phi_{j}}{\phi_{j}+1}\right)=-\log\frac{\phi_{j}}{\phi_{j}+1}$$

The mutual entropy of reverse signal transduction is also given by the entropy *h*_−*j*_^0^ = *h_j_*^0^ = −*q_j_*log*q_j_* − q_j_*logq_j_* and mutual entropy as *h*_−*j*_^0^ − *h*(−*j* − 1|−*j*) (Figure 1). The following function, *U*_−*j*_ for the reverse transduction using the undetermined parameter *λ*′, is maximised as follows:

(30) $$U_{-j}=-q_{j}\log q_{j}-q_{j}*\log q_{j}*-\xi_{-j}q_{j}+\lambda^{\prime }\left[q_{j}+q_{j}*\right]$$

In above, *ξ*_−*j*_ = −*ϕ*_−*j*_ log*ϕ*_−*j*_ − (1 − *ϕ*_−*j*_) log (1 − *ϕ*_−*j*_), and *ϕ*_−*j*_ denotes the noise occurrence probability in the reverse cascade. Then:

(31) $$\frac{\partial }{\partial q_{j}}U_{-j}=-\log q_{j}-1+\lambda^{\prime }-\xi_{-j}$$

(32) $$\frac{\partial }{\partial q_{j}*}U_{-j}=-\log q_{j}*-1+\lambda^{\prime }$$

Setting the right-hand side of Equations (31) and (32) to zero, and eliminating *λ*′, we have:

(33) $$\log\frac{q_{j}}{q_{j}*}=\log\frac{p_{j}}{p_{j}*}=-\xi_{-j}=\xi_{j}$$

Accordingly, the channel capacity *C*_−*j*_ is given by:

(34) $$\begin{array}{l} C_{-j}≜h_{-j}{}^{\max}≜\left(-\frac{\phi_{j}}{\phi_{j}+1}\log\frac{\phi_{j}}{\phi_{j}+1}-\frac{1}{\phi_{j}+1}\log\frac{1}{\phi_{j}+1}-\xi_{-j}\frac{1}{\phi_{j}+1}\right)\\ =\left(-\frac{\phi_{j}}{\phi_{j}+1}\log\frac{\phi_{j}}{\phi_{j}+1}-\frac{1}{\phi_{j}+1}\log\frac{1}{\phi_{j}+1}+\log\phi_{j}\frac{1}{\phi_{j}+1}\right)\\ =-\log\frac{1}{\phi_{j}+1} \end{array}$$

The channel capacity of the *j^th^* cycle step is defined and calculated as follows:

(35) $$h_{j}=C_{-j}-C_{j}=\log\phi_{j}=\xi_{j}=\log\frac{p_{j}}{p_{j}*}$$

Thus, we can obtain the mutual entropy as entropy difference in Equation (15).

## 5. Szilard Engine Chain as a BRC Model

Subsequently, let us consider that phosphorylation and dephosphorylation reactions form a cycle reaction that simultaneously activates the next cycle reaction in a BRC. A cycle reaction of individual step can be modelled as a *Szilard* engine, which may serve as a model of the conversion system [17]. The Szilard engine was established by Leo Szilard considering Maxwell’s demon paradox [31,32]. In the engine model, Maxwell’s demon, which is a feedback controller, utilises the position information of a single gas particle in a box that contacts with a heat bath. As an initial state, the boundary is inserted to a room at the middle position such that the controller can determine whether a single gas particle is in the left space or in the right space of the room. The information gained by the controllers is equal to one bit (i.e., left or right). In the case of the particle in the left, let the boundary be quasi-statically moved in the right orientation for recovery of the full volume of the room. In both cases, the particle isothermally expands with the movement of the boundary back to its original full volume. The extracted work is equal to *k_B_T*ln2. This process is equivalent to the system, in which the feedback controller transforms the gained information into the actual expansion work. The feedback controller system has been produced in the actual experimental study [33,34]. Thereby, let us consider the feedback controller is informed whether the signalling molecule is an active or inactive type in place of measuring the particle position.

**Figure 2:**
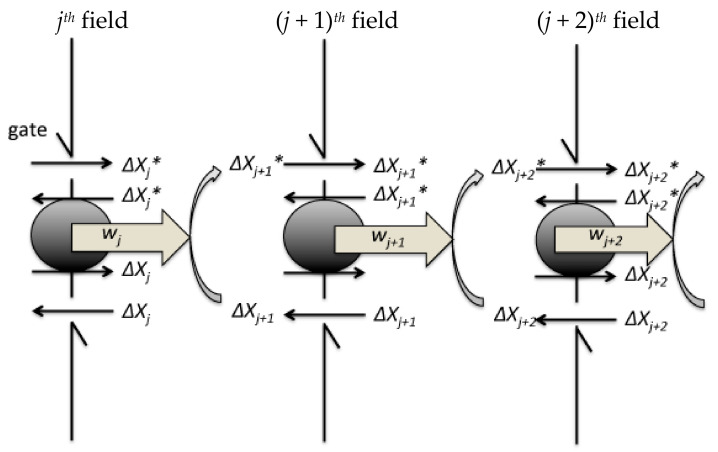
Schematic showing a Szilard engine chain. The feedback controller measures the changes in concentration of signalling molecules. For the signal transduction, the controller opens the gate of the hypothetical boundary. The grey circles on the boundary represent the exchanger between Δ*X_j_*_+1_ and Δ*X_j_*_+1_*. The *j^th^* field recovers to the initial state.

As reported previously [17,19], the BRC for modelling can be divided into *n* number of hypothetical compartment fields corresponding to the individual *j^th^* steps (1 ≤ *j* ≤ *n*) that corresponds to a single Szilard engine. The diffusion rate of signaling molecule is sufficiently low because of its high molecular weight and they are hypothesised to be localized in the compartment fields. Each field contains all *X_j_*_+1_* and *X_j_*_+1_ species (1 ≤ *j* ≤ *n* − 1), with the concentrations identical to those of *X_j_*_+1_*^st^ and *X_j_*_+1_*^st^*, respectively, at the steady state. The feedback controller has the potential to recognise the molecule concentration. Subsequently, the controller selects *X_j_*_+1_* or *X_j_*_+1_ for its transfer (Figure 2). The steps are summarised as follows when BRC proceeds:

(i) When the signal transduction initiates, the controller measures the changes in the concentration of the active molecule *X_j_*_+1_* and *X_j_*_+1_ in the *j^th^* field.
(ii) At the *j^th^* step in the signalling cascade, the feedback controller introduces Δ*X_j_*_+1_* of *X_j_*_+1_* to the (*j* + 1)*^th^* field from the *j^th^* field by opening the forward gate on the boundary in the *j^th^* field to the (*j* + 1)*^th^* field. Simultaneously, the controller introduces Δ*X_j+1_* of *X_j+1_* to the (*j* + 1)*^th^* field from the *j^th^* field by opening the back gate on the boundary.
(iii) Subsequently, *X_j_*_+1_* can flow back with the forward transfer of *X_j_*_+1_ from the (*j* + 1)*^th^* field to the *j^th^* field because of the entropy difference (see Equation (13)). *X_j_*_+1_ can also flow back with the backward transfer of *X_j_*_+1_ from the (*j* + 1)*^th^* field to the *j^th^* field because of the concentration gradient.
(iv) In (iii), Δ*X_j_*_+1_* and Δ*X_j_*_+1_ can *quasi-statically* rotate the exchange machinery on the hypothetical partition between the *j^th^* and (*j* + 1)*^th^* fields, which has the ability to extract chemical work equivalent to *w*_*j*+__1_ = *k_B_Th_j+1_*.
(v) As the next step, *w*_*j*+__1_ is linked to the modification of *X_j_*_+2_ into *X_j_*_+2_*, which further causes the concentration difference of *X_j_*_+2_* introduced by the feedback controller from the (*j* + 1)*^th^* field to the (*j* + 2)*^th^* field. The next step proceeds as aforementioned in (ii) to (iii).

Accordingly, replacing the suffix *j* + 1 by *j* for simplification, the chemical work *w****_j_*** extracted from the *j^th^* Szilard engine is given using the mutual entropy *h_j_* informed to the controller whether the signalling molecules increase or decrease according to Equations (15) and (35) [12,13,14,18,19]:

(36) $$w_{j}=k_{B}Th_{j}\Delta X_{j}*=k_{B}T\Delta X_{j}*\log\frac{p_{j}}{p_{j}*}$$

## 6. Conservation of the Average Entropy Production

Next, let us review the optimized coding way for maximizing the signal event number for a given duration in this binary coding model of signal transduction in a nonequilibrium steady system. First, the duration of signal transduction is defined in consideration of the signal orientation (i.e., forward *τ_j_* and backward *τ*_−*j*_). Positive and negative values are assigned to *τ_j_* and *τ*_−*j*_ for distinction of the signal direction. *τ_j_* represents the duration of the tentative increase in the active molecule X_j_*, whilst *τ*_−*j*_ represents the duration to the recovery to the initial state. The step cycle duration is represented by *τ_j_* − *τ*_−*j*_.

The average entropy production *ζ_j_* and *ζ*_−*j*_ during the signal transduction are defined during *τ_j_* − *τ*_−*j*_ and the average entropy production rate (AEPR) is defined using a bracket < > as:

(37) $$\langle \zeta_{j}\rangle ≜\frac{1}{\tau_{j}-\tau_{-j}}\int_{0}^{\tau_{j}-\tau_{-j}}\zeta_{j}\left(s_{j}\right)ds_{j}$$

(38) $$\langle \zeta_{-j}\rangle ≜\frac{1}{\left|\tau_{j}-\tau_{-j}\right|}\int_{0}^{\left|\tau_{j}-\tau_{-j}\right|}\zeta_{j}\left(s_{j}\right)ds_{j}$$

where, *s_j_* is an arbitrary parameter representing the progression of a reaction event [35]. The transitional probability *p* (*j* + 1|*j*) is the probability of the (*j* + 1)*^th^* step given the *j^th^* step during *τ_j_*, and *p* (*j*|*j* + 1) is the transitional probability of the *j^th^* step given the (*j* + 1)*^th^* step during *τ*_−*j*_. The AEPR <*ζ_j_* > during the signal transduction from the *j*^th^ to the (*j* + 1)*^th^* field is given according to fluctuation theorem (FT) at the steady state:

(39) $$\underset{\tau_{j}-\tau_{-j}\to \infty }{\lim}\frac{1}{\tau_{j}-\tau_{-j}}\log\frac{p\left(j+1|j\right)}{p\left(j|j+1\right)}=\langle \zeta_{j}\rangle$$

The AEPR <*ζ*_−*j*_> from the (*j* + 1)*^th^* to the *j^th^* field is given:

(40) $$\underset{\left|\tau_{-j}-\tau_{j}\right|\to \infty }{\lim}\frac{1}{\left|\tau_{-j}-\tau_{j}\right|}\log\frac{p\left(j|j+1\right)}{p\left(j+1|j\right)}=\langle \zeta_{-j}\rangle$$

The following equation is given using signal current density *c_j_* in (17) [19,35]:

(41) $$\underset{\tau_{j}-\tau_{-j}\to \infty }{\lim}\frac{1}{\tau_{j}-\tau_{-j}}\log\frac{p\left(j+1|j\right)}{p\left(j|j+1\right)}=\frac{c_{j}}{k_{B}T\left(\tau_{j}-\tau_{-j}\right)}\Delta X_{j}*$$

Substituting the right side of Equation (17) into the right side of (41), an important result is given [19]:

(42) $$\underset{\tau_{j}-\tau_{-j}\to \infty }{\lim}\frac{1}{\tau_{j}-\tau_{-j}}\log\frac{p\left(j+1|j\right)}{p\left(j|j+1\right)}=\underset{\tau_{j}-\tau_{-j}\to \infty }{\lim}\frac{1}{\tau_{j}-\tau_{-j}}\log\frac{p_{j}}{p_{j}*}$$

When the signal even number is maximised, the logarithm of the selection probability is described simply using the average entropy production rate *β* independent of the step number according to previous reports [17,18,19,20]:

(43) $$-\log p_{j}=\beta \tau_{j}$$

(44) $$\log p_{j}*=\beta \tau_{-j}$$

This is one type of entropy coding. Substitution of the right sides of Equations (43) and (44) into (42) gives:

(45) $$\underset{\tau_{j}-\tau_{-j}\to \infty }{\lim}\frac{1}{\tau_{j}-\tau_{-j}}\log\frac{p\left(j+1|j\right)}{p\left(j|j+1\right)}=\underset{\tau_{j}-\tau_{-j}\to \infty }{\lim}\beta \frac{-\tau_{j}-\tau_{-j}}{\tau_{j}-\tau_{-j}}\sim -\beta$$

Likewise,

(46) $$\underset{\left|\tau_{j}-\tau_{-j}\right|\to \infty }{\lim}\frac{1}{\left|\tau_{j}-\tau_{-j}\right|}\log\frac{p\left(j+1|j\right)}{p\left(j|j+1\right)}=\underset{\left|\tau_{j}-\tau_{-j}\right|\to \infty }{\lim}\beta \frac{-\tau_{j}-\tau_{-j}}{\tau_{j}-\tau_{-j}}\sim \beta$$

**Figure 3:**
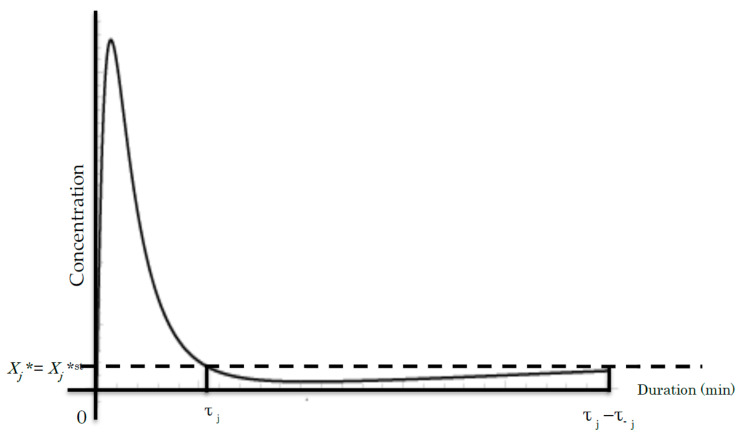
A common time course of the *j^th^* cycle showing the concentration of X_j_* during phosphorylation [36,37]. The vertical axis represents the concentration of X_j_*. The horizontal axis denotes the duration (min or time unit). *τ_j_* and *τ*_−*j*_ denote the duration of the *j^th^* step and the reversible −*j^th^* step, respectively. Line X_j_* = X_j_*^st^ denotes the X_j_* concentration at the initial steady state before the beginning of the signal event.

We used *τ_j_* << *τ*_−*j*_ as shown in Figure 3 in (45) and (46) and sufficient long duration of the whole signal transduction according to experimental data [23,36,37]. The dephosphorylation of signaling molecule X_j_* takes a significantly longer time, *τ*_−*j*_. Subsequently, Equations (39), (40), (45) and (46) provide:

(47) $$\beta =-\langle \zeta_{j}\rangle =\langle \zeta_{-j}\rangle \equiv \langle \zeta \rangle$$

In summary, we obtained the following result from Equations (43)–(47):

(48) $$-\log p_{j}=\langle \zeta \rangle \tau_{j}$$

(49) $$\log p_{j}*=\langle \zeta \rangle \tau_{-j}$$

Equations (48) and (49) implies the integration of information entropy, code length, and thermodynamic AEPR. In these equations, step numbers *j* and −*j* in *ζ_j_* and *ζ*_−*j*_ were omitted because *ζ_j_* and *ζ*_−*j*_ are independent of the step number. Thus, the theoretical basis of the consistency of the average entropy production rate can be obtained.

The chemical extracted average chemical work <*w_j_*> in Equation (36) from (i) to (iv) in Section 5 is calculated as follows using Equations (15), (36), (48) and (49) [18]:

(50) $$\langle w_{j}\rangle =k_{B}TH_{j}=\overset{}{\underset{}{\int }}k_{B}T\log\frac{p_{j}}{p_{j}*}dX_{j}*=k_{B}T\Delta X_{j}*\langle \zeta \rangle \left(\tau_{j}-\tau_{-j}\right)$$

The summation of the right side of (50) gives the total work:

(51) $$\langle w\rangle ≜k_{B}T\overset{n}{\underset{j=1}{\sum }}\langle \zeta \rangle \left(\tau_{j}-\tau_{-j}\right)\Delta X_{j}{}^{*}=k_{B}TH$$

with

(52) $$H≜\langle \zeta \rangle \overset{n}{\underset{j=1}{\sum }}\left(\tau_{j}-\tau_{-j}\right)\Delta X_{j}*=\overset{n}{\underset{j=1}{\sum }}\sigma_{j}\Delta X_{j}*$$

and

(53) $$\sigma_{j}≜\langle \zeta \rangle \left(\tau_{j}-\tau_{-j}\right).$$

Here, *σ_j_* stands for the entropy production during *τ_j_* − *τ*_−*j*_ at the *j^th^* step.

## 7. Conclusions

Signal transduction is an important research topic in life science, but quantitatively evaluating data remains difficult. This review pointed out the possibility of quantitative signalling to life scientists. The current review can be summarised in the following points:

(i) The BRC can be expressed by a kind of binary code system consisting of two types of signalling molecules: activated and inactivated.
(ii) The individual reaction step of the BRC can be thought of as a cycle of a Szilard engine chain, in which the process of repeats of signalling molecule activation/inactivation.
(iii) The average entropy production rate is consistent during BRC.
(iv) The signal transduction amount can be calculated through the BRC.

The chain of Szilard engines is a useful model to show how signal transduction in one step induces signal transduction in the next step, in which a series of chains is formed. The most important point of this model is to directly give the signal transduction amount by the exchange work according to Equation (3). The currently introduced chain illustrates that the feedback controller transfers signal molecules based on the measurement of the increase and decrease of the signal molecule. Subsequently, the exchanger molecule on the boundary between the steps can extract work between because of the entropy gradient consisting of the two types of signalling molecules. In this way, the signal transduction amount can be clearly quantified by the combination of chemical work.

Herein, let us consider the calculation of the entropy production based on the kinetics of the activation of signalling molecules according to (5). The signalling system is contacted with a chemical bath outside the system that provides ATP. The transitional rate from the *j^th^* step to the (*j* + 1)*^th^* step, *v_j_*, obtained using the kinetic coefficient *k_j_* for the *j^th^* step as follows:

(54) $$v_{j}=k_{j}AX_{j}*X_{j+1}$$

The transitional rate from the (*j* + 1)*^th^* step to the *j^th^* step, *v*_−*j*_, which is equal to the demodification (dephosphorylation) of the backward signal transduction, is given using the kinetic coefficient *k*_−*j*_ for the −*j^th^* step:

(55) $$v_{-j}=k_{-j}Ph_{j+1}X_{j+1}*$$

where, *k_j_* and *k*_−*j*_ represent the kinetic coefficients. The signal transduction system remains at a detailed balance around the steady state, the homeostatic point:

(56) $$p\;(j\left|j+1)v_{-j}=p(j+1\right|j)v_{j}$$

Combining Equations (47), (54)–(56), we obtain the following from FT:

(57) $$\begin{array}{l} \underset{\tau_{j}-\tau_{-j}\to \infty }{\lim}\frac{1}{\tau_{j}-\tau_{-j}}\log\frac{p(j+1|j)}{p(j|j+1)}=\underset{\tau_{j}-\tau_{-j}\to \infty }{\lim}\frac{1}{\tau_{j}-\tau_{-j}}\log\frac{k_{j}AX_{j}*X_{j+1}}{k_{-j}Ph_{j+1}X_{j+1}*}\\ ==\underset{\tau_{j}-\tau_{-j}\to \infty }{\lim}\frac{1}{\tau_{j}-\tau_{-j}}\left(\log\frac{k_{j}AX_{j}*}{k_{-j}Ph_{j+1}}+\log\frac{p_{j+1}}{p_{j+1}*}\right)\\ ≃\underset{\tau_{j}-\tau_{-j}\to \infty }{\lim}\frac{1}{\tau_{j}-\tau_{-j}}\log\frac{p_{j+1}}{p_{j+1}*}=-\langle \zeta \rangle \end{array}$$

Above result contains Equation (42). Therefore, for sufficient long duration *τ_j_* − *τ*_−*j*_:

(58) $$\log\frac{p(j+1|j)}{p(j|j+1)}=\log\frac{k_{j}AX_{j}*X_{j+1}}{k_{-j}Ph_{j+1}X_{j+1}*}≃-\langle \zeta \rangle \left(\tau_{j}-\tau_{-j}\right)=-\sigma_{j}.$$

Using the concentration of the active signalling molecules at the steady state, *X*_*j*+__1_^st^*, we have:

(59) $$X_{j+1}*=X_{j+1}{}^{st}*+\Delta X_{j+1}*$$

Substitution of Equation (59) into Equation (58) produces:

(60) $$\log\frac{k_{j}AX_{j}*X_{j+1}}{k_{-j}Ph_{j+1}X_{j+1}*}=-\sigma_{j}+\log\frac{k_{j}AX_{j}{}^{st}*X^{st}{}_{j+1}}{k_{-j}Ph_{j+1}X_{j+1}{}^{st}*}$$

Here, the entropy production *σ_j_* in (53) is defined in the *j^th^* step:

(61) $$-\sigma_{j}=k_{B}T\log\left(\frac{1+\Delta X_{j+1}/X^{st}{}_{j+1}}{1+\Delta X_{j+1}*/X^{st}{}_{j+1}*}\right).$$

In Equation (61), the fluctuation of X_j_* is negligible during signal transduction relative to Δ*X*_*j*+__1_ and Δ*X*_*j*+__1_*** according to experimental data (36). The sum of the concentrations of *X*_*j*+__1_ and *X*_*j*+__1_*** is equal to the total concentration *X_j_*_+1_^0^ that is kept constant because the signal transduction rate is significantly greater than the production of signalling molecular proteins. Then:

(62) $$X_{j+1}+X_{j+1}*=X_{j+1}{}^{0}=const.$$

Equations (54), (55) and (62) give the concentrations at steady state:

(63) $$X_{j+1}{}^{st}=\frac{k_{-j}Ph_{j+1}p(j|j+1)}{k_{-j}Ph_{j+1}p(j\left|j+1)+k_{j}p(j+1\right|j)A}X_{j+1}{}^{0}$$

(64) $$X_{j+1}{}^{st}*=\frac{k_{j}p(j+1|j)A}{k_{-j}Ph_{j+1}p(j\left|j+1)+k_{j}p(j+1\right|j)A}X_{j}{}^{0}$$

*Ph_j_*_+1_ signifies the phosphatase concentration in the *j^th^* step. The fluctuation of the transmitted information is described as follows using an integral form of Equation (61):

(65) $$\begin{array}{l} -\sigma_{j}=\overset{\tau_{j}-\tau_{-j}}{\underset{0}{\int }}\log\left(\frac{1+\Delta X_{j+1}/X^{st}{}_{j+1}}{1+\Delta X_{j+1}*/X^{st}{}_{j+1}*}\right)\frac{\Delta X_{j+1}*}{\Delta s_{j}}ds_{j}\\ =\overset{\tau_{j}-\tau_{-j}}{\underset{0}{\int }}\frac{X_{j+1}{}^{0}}{X_{j+1}{}^{st}*X_{j+1}{}^{st}}\frac{\Delta X_{j+1}*}{\Delta s_{j}}ds_{j}=\overset{\tau_{j}-\tau_{-j}}{\underset{0}{\int }}\frac{X_{j+1}{}^{0}}{X_{j+1}{}^{st}*X_{j+1}{}^{st}}\frac{dX_{j+1}*}{dA}\frac{dA}{ds_{j}}ds_{j} \end{array}$$

We used the approximation log (1 + *x*) ~ *x* in the logarithmic term in (65). A simple calculation of Equations (63) and (64) gives:

(66) $$\frac{dX_{j+1}*}{dA}=\frac{1}{A}\frac{X_{j+1}{}^{st}*X_{j+1}{}^{st}}{X_{j+1}{}^{0}}$$

Then, substitution of Equation (66) into Equation (65) gives:

(67) $$\begin{array}{l} -\sigma_{j}=\int_{A_{ji}}^{A_{jf}}\frac{1}{A}dA={\left[\log A\right]}_{A_{ji}}^{A_{jf}}\\ =\log\frac{A_{jf}}{A_{ji}}=\log\frac{A_{ji}-\Delta A_{j}}{A_{ji}}\approx -\frac{\Delta A_{j}}{A_{ji}} \end{array}$$

with

(68) $$A_{jf}=A_{ji}-\Delta A_{j}$$

*A_jf_* and *A_ji_* signify the local concentration of the mediator ATP at the initial and final state, respectively, at the *j^th^* step. Δ*A_j_* signifies the concentration change of ATP at the *j^th^* step. Thus, the total entropy production *σ* is simply given as follows:

(69) $$\sigma ≜\overset{n}{\underset{j=1}{\sum }}\sigma_{j}=-\overset{n}{\underset{j=1}{\sum }}\log\frac{A_{jf}}{A_{ji}}=-\log\frac{A_{1i}-\overset{n}{\underset{j=1}{\sum }}\Delta A_{j}}{A_{1i}}≃\frac{\overset{n}{\underset{j=1}{\sum }}\Delta A_{j}}{A}.$$

In above, we used the approximation log (1 + *x*) ~ *x* again and set *A*_1*i*_ equal to the initial concentration of ATP, *A*. Thus, ATP is the *mediator* of signal transduction. In an actual experiment, rigorously measuring the concentration change of ATP at individual signal steps is difficult because ATP is consumed in a variety of reactions as a basic metabolite for cell activity. Alternatively, the ratio Δ*X*_*j*+__1_/Δ*X*_*j*+__1_*^st^* is negligible during signal transduction according to experimental data [36], we have from (61):

(70) $$-\sigma_{j}=\int_{0}^{\tau_{j}-\tau_{-j}}\log\left(1+\Delta X_{j+1}*\left(s_{j}\right)/\Delta X_{j+1}{}^{st}\;*\right)ds_{j}$$

or

(71) $$-\langle \zeta \rangle =\frac{-\sigma_{j}}{\tau_{j}-\tau_{-j}}=\frac{1}{\tau_{j}-\tau_{-j}}\int_{0}^{\tau_{j}-\tau_{-j}}\log\left(1+\Delta X_{j+1}*\left(s_{j}\right)/\Delta X_{j+1}{}^{st}\;*\right)ds_{j}$$

As aforementioned, the right side of Equation (71) indicates that AEPR <*ζ*> is consistent during the cascade. Accordingly, the measurement of AEPR will provide an evidence of its consistency during the signaling cascade. In this manner, the rigorous measurement of the concentration change of active signaling molecule may provide more direct evidence in the presented theory.To date, experimental data have demonstrated that the time course of increase in active signaling molecules shows a similar time course plot, as shown in Figure 3, suggesting the consistency of the AEPR [36,37].

Further study is required to prove which signal transduction strategy a biological system will select. For example, the cell system may select a strategy to maximise signal event number during a given duration by application of non-redundant signal system; in contrast, accuracy of the signal transduction may be prioritized by application of redundant signal system. The strategy chosen for signal cascade by the cell system will likely be determined experimentally. The cost-performance of metabolomics substance tradeoffs for cellular regulatory functions and information processing will been argued by evaluation of recent experimental data [23,24,25,26]. By measuring the consumption of metabolite, Luo et al. were successful in their estimation of biological information [38]. The relationship between the ATP concentration in cellular tissues and information transmission has been vigorously studied in the analysis of nerve excitement transmission [39], and this review may suggest implications for quantitative information transmission.

The discussion developed herein has some limitations; hence, we would like to mention it at the end. A detailed balancing between modification and demodification is assumed at each step for the application of FT. Therefore, the current discussion is also possible only when the distance from the detailed balance is not great [17,18,19,20]. This also depends on how we consider the range of FT application or Jarzynski equality. FT has been applied to study a non-equilibrium system [28,29,40,41], limit cycle [42], molecular machines [43], and biological phenomenon [44], including membrane transport [45], molecular motor activity [46], and RNA folding [47]. The adaptation and extension of the current discussion to the non-linear phenomenon [48,49] and far from steady state or active matters will be the next theoretical subjects. However, at the least, interpreting the signal cycle as a Szilard engine is considered as an effective idea for thought of experiments, and a chain of the engines will serve as an actual BRC.

In conclusion, the information thermodynamics approach described herein provides a framework for the analysis of signal transduction BRC. This theoretical approach appears suitable for the identification of novel active signalling cascades among response cascades in which AEPR is consistent through the given cascade. This review presents that the binary coding system and the Szilard engine chain model may be the theoretical basis of computation of the channel capacity of BRC.
